# Upregulation of Runt related transcription factor 1 (RUNX1) contributes to tendon–bone healing after anterior cruciate ligament reconstruction using bone mesenchymal stem cells

**DOI:** 10.1186/s13018-022-03152-y

**Published:** 2022-05-13

**Authors:** Kai Kang, Qian Geng, Lukuan Cui, Lijie Wu, Lei Zhang, Tong Li, Qian Zhang, Shijun Gao

**Affiliations:** The Second Department of Joint Surgery, Third Hospital of Hebei Medical University, 139 Ziqiang Road, Shijiazhuang, 050051 Hebei People’s Republic of China

**Keywords:** Runt related transcription factor 1, Anterior cruciate ligament reconstruction, Tendon–bone healing, Bone mesenchymal stem cells

## Abstract

**Background:**

Anterior cruciate ligament (ACL) injury could lead to functional impairment along with disabilities. ACL reconstruction often fails owing to the regeneration failure of tendon–bone interface. Herein, we aimed to investigate the effects of Runt related transcription factor 1 (RUNX1) on tendon–bone healing after ACL reconstruction using bone mesenchymal stem cells (BMSCs).

**Methods:**

BMSCs were isolated from the marrow cavity of rat femur, followed by the modification of RUNX1 with lentiviral system. Then, an ACL reconstruction model of rats was established with autografts.

**Results:**

Results of flow cytometry exhibited positive-antigen CD44 and CD90, as well as negative-antigen CD34 and CD45 of the BMSCs. Then, we found that RUNX1-upregulated BMSCs elevated the decreased biomechanical strength of the tendon grafts after ACL reconstruction. Moreover, based on the histological observation, upregulation of RUNX1 was linked with better recovery around the bone tunnel, a tighter tendon–bone interface, and more collagen fibers compared to the group of BMSCs infected with LV-NC. Next, RUNX1-upregulated BMSCs promoted osteogenesis after ACL reconstruction, as evidenced by the mitigation of severe loss and erosion of the cartilage and bone in the tibial and femur area, as well as the increased number of osteoblasts identified by the upregulation of alkaline phosphatase, osteocalcin, and osteopontin in the tendon–bone interface.

**Conclusion:**

Elevated expression of RUNX1 contributed to tendon–bone healing after ACL reconstruction using BMSCs.

## Introduction

Tendons and ligaments attach to bone through a transitional fibrocartilage tissue, which is known as the tendon–bone interface [1]. This transitional tissue consists of four zones as follows, tendon, uncalcified fibrocartilage, calcified fibrocartilage, and bone [1]. Tendon–bone interface is mainly responsible for the transmission of force between tendon and bone [2]. The transitional tissues could improve the connected strength and protect against the damage induced by excessive tension [3]. Due to the complex composition and organization, tendon–bone interface is hard to regenerate during healing of injury [4, 5]. Thus, anterior cruciate ligament (ACL) reconstruction, the most common tendon–bone healing surgery, often fails owing to the regeneration failure of tendon–bone interface [4]. ACL injury may lead to functional impairment along with disabilities as a result of knee joint laxity, decreased quadriceps strength, meniscal damages, as well as poor knee joint loading [6]. Thus, there is an urgent need to improve the healing of tendon–bone interface to achieve the development of the ACL reconstruction.

Notably, bone mesenchymal stem cells (BMSCs) has been testified to contribute to ligament regeneration and graft-bone healing after ACL reconstruction with silk-collagen scaffold [7]. BMSCs are a kind of pluripotent cells and become an essential cell source for musculoskeletal tissue engineering repair [8]. The differentiation of BMSCs into osteoblasts and chondrocytes can promote tendon–bone healing [9]. These studies indicate the protective role of BMSCs in tendon–bone healing after ACL reconstruction. Moreover, mesenchymal stem cells products are safe in the short- and mid-term, but studies with long follow-up are deficient [10, 11]. Although the number of clinical researches is low, there is great therapeutic potential of the stem-cell products [10, 12]. Whereas regenerative technologies still need to be optimized.

Runt related transcription factor 1 (RUNX1), also known as AML1 and cbfa2, is a member of the Runt-related transcription factor family [13]. RUNX1 is widely reported to involve in the hematopoietic development, and genetic ablation of RUNX1 causes embryonic lethality [14, 15]. Recently, RUNX1 is found to be linked with fracture healing. Specifically, the expression of RUNX1 is detected in osteoblast progenitors, pre-osteoblasts, and mature osteoblasts [16]. RUNX1 facilitates bone formation by promoting both chondrogenesis and osteogenesis, and RUNX1 chondrocyte-specific knockout mouse exhibited skeletal malformation and dwarfism [17]. Tang et al. has testified that RUNX1 upregulation ameliorates bone loss in ovariectomy-induced osteoporosis [16]. These studies indicated the potential of RUNX1 in the promotion of bone formation. Moreover, RUNX1 up-regulation induces BMSCs to undergo chondrogenic differentiation [18]. Luo et al. have demonstrated that RUNX1 regulates osteogenic differentiation of BMSCs via repressing the adipogenesis by the Wnt/β-catenin pathway [19]. However, the role of RUNX1 in BMSCs is unclear regarding tendon–bone healing after ACL reconstruction.

In the current study, we aimed to investigate the effects of RUNX1 on tendon–bone healing in tendon–bone healing after ACL reconstruction employing BMSCs in a rat model.

## Materials and methods

### Ethics statement

Animal experiments were approved by the Medical Ethic Committee of Third Hospital of Hebei Medical University (Z2020-009-1) in strict accordance with the standard of The Guideline for the Care and Use of Laboratory Animals.

### Experimental animals

Ten-week-old Sprague Dawley rats (male) were purchased from Liaoning Changsheng biotechnology (Benxi, China). Rats were housed in cages (3–4 animals per cage) with food and water ad libitum in a temperature-controlled room (22 ± 1 °C) under a 12/12 h light–dark cycle.

### Isolation, culture, and identification of BMSCs

After sacrifice, the femur of rats was collected under aseptic condition. The muscle and connective tissues were removed from the femur, and then BMSCs were harvested by flushing the marrow cavity with MEM medium (Solarbio, Beijing, China). After centrifugation at 150*g* for 10 min, BMSCs were collected and washed with phosphate buffer saline (PBS) for triplicate. Subsequently, cells were maintained in MEM medium supplemented with 10% fetal bovine serum at 37 °C with 5% CO_2_ for 24 h. BMSCs were washed by PBS to remove the exfoliated cells, and the culture medium was replaced for further culture.

After digestion, BMSCs were collected and incubated with antibodies FITC-conjugated anti-CD45 (0.25 μg per million cells in 100 μL volume; MultiSciences Biotech, Hangzhou, China) for 30 min and PE-conjugated anti-CD90 (0.03 μg per million cells in 100 μL volume; Biolegend, San Diego, California, USA) for 20 min in the dark. For CD44 and CD34, collected cells were incubated with anti-CD44 (0.2 μg per million cells in 100 μL volume; Proteintech, Wuhan, China) for 45 min and anti-CD34 (1 μg per million cells in 100 μL volume; Santa Cruz, CA, USA) for 20 min, followed by the incubation with FITC-conjugated goat anti-mouse or goat anti-rabbit IgG (diluted 1:500; Abcam, Cambridge, UK.) for 30 min at 4 °C preventing from the light. Next, samples were analyzed using a NovoCyte flow cytometer (Aceabio Company, Calif, USA).

### Lentivirus vector construction and cell infection

To construct the RUNX1-upregulated vectors, the cDNA sequences of RUNX1 were cloned into the lentiviral vector pLVX-AcGFP1-N1 (Fenghuishengwu, Hunan, China). Then, HEK-293T cells were co-transfected with lentiviral vector and helper vector (pSPAX2 and pMD2.G; Fenghuishengwu) by employing the Lipofectamine 2000 (Invitrogen, Carlsbad, California, USA) following users’ protocol. After transfection for 48 h, lentiviral supernatant was collected employing centrifugation at 956*g* for 45 min, followed by passing through a 45 μm filter. Subsequently, BMSCs were infected with the lentiviral particles at the optimal multiplicity of infection of 80.

### Quantitative real-time polymerase chain reaction (qRT-PCR)

Total RNA isolation was carried out from infected cells by using the TRIpure (BioTeke, Beijing, China) according to the manufacturer’s protocol. Then, the RNA was reverse transcribed into cDNA with the BeyoRT II M-MLV reverse transcriptase (Beyotime, Shanghai, China). Subsequently, amplification was performed by employing the 2 × Taq PCR MasterMix (Solarbio, Shanghai, China) and SYBR Green (Solarbio). The expression of RUNX1 was calculated with 2^−ΔΔCt^ method. β-actin was employed as the internal control. The sequences of primer were as follows. Forward primer: 5′-GACCCTGCCCATCGCTTTC-3′; Reverse primer: 5′-AATCTCGCCACTTGGTTCTTC-3′.

### Western blot

Total protein isolation was carried out from infected cells by employing the phenylmethanesulfonyl fluoride (Beyotime) mixed with RIPA lysis solution (Beyotime). Protein concentration was measured by the BCA Protein Assay Kit (Beyotime). 25 μg protein was separated by sodium dodecyl sulfate polyacrylamide gel electrophoresis and then transferred to polyvinylidene fluoride membranes (Merk Millipore, Billerica, MA, USA). After blocked with 5% skimmed milk, membranes were incubated with the antibodies against RUNX1 (1:400; Affinity, Changzhou, China) or β-actin (1:1000; Santa Cruz) at 4 °C overnight and then with the secondary antibodies horseradish peroxidase-conjugated goat anti-rabbit IgG (1:5000; Beyotime) or goat anti-mouse IgG (1:5000; Beyotime). Protein blot bands were visualized with WD-9413B gel imaging system (Liuyi biotechnology, Beijing, China).

### ACL reconstruction model

After 1 week of acclimation, rats were randomly divided into four groups: sham, model, model + BMSCs-LV-NC, model + BMSCs-LV-RUNX1. The ACL reconstruction model was established according to the procedures described previously [20]. After anaesthesia, an anteromedial incision was made, and a medial parapatellar arthrotomy was employed to expose and resect the native ACL. The tendon was harvested, and femoral tunnels of 1.5 mm in diameter were created through the femur and tibia around the insertion site of the native ACL. The graft was routed through the bone tunnels to replace the ACL. For the groups of model + BMSCs-LV-NC and model + BMSCs-LV-RUNX1, BMSCs (1 × 10^6^) infected with LV-NC or LV-RUNX1 mixed with 0.2 mL of fibrin glue were injected to the periphery of tunnel surrounding the tendon graft. For the model group, only 0.2 mL of fibrin glue without BMSCs was injected around the bone tunnels as described above. For the sham group, only an anteromedial incision was made without the resection of native ACL. Twelve weeks after reconstruction, knees were harvested for further experiments.

### Biomechanical analysis

Biomechanical properties of the healing interface were measured following the previous study [21]. The femur-ACL graft-tibia complexes were mounted onto a BOSE Electroforce 3200 biomaterials test instrument (Eden Prairie, MI, USA). The sutures and scar tissues were removed from the femoral tunnel. After cyclic preconditioning at the maximum displacement of 0.5 mm, the construct was loaded at a displacement rate of 20 mm/min. The load to failure was recorded, and the stiffness of the femur-ACL graft-tibia complex was calculated from the liner portion of the load–displacement curve [22].

### Histological analysis

Paraffin-embedded sections of tendon–bone graft (5 μm) were deparaffinized in xylene, and rehydrated in an ethanol gradient with distilled water. Haematoxylin and eosin (H&E) and Masson’s trichrome staining were carried out to evaluate the histopathological changes. Sections were imaged using BX53 microscope at 400× magnification (Olympus, Tokyo, Japan).

For immunohistochemistry staining, the activity of endogenous peroxidase was quenched by incubation with 3% hydrogen peroxide for 15 min. After blocked with 1% bovine serum albumin, sections were incubated with primary antibodies against osteocalcin (OCN; 1:100; Affinity) and osteopontin (OPN; 1:100; Affinity) at 4 °C overnight, followed by the incubation with second antibodies horseradish peroxidase-conjudated goat anti-rabbit IgG (1:500; ThermoFisher Scientific, Pittsburgh, PA, USA) at 37 °C for an hour. Subsequently, sections were counterstained with hematoxylin (Solarbio) for 3 min. Images were captured with BX53 microscope at 800× magnification.

The expression of alkaline phosphatase (ALP) in the tendon–bone interface was measured by using the ALP kit (Leagene Biotech, Beijing, China) in accordance with the manufacturers’ instructions.

### Micro-CT assessment

Bone and cartilage part of the specimens was scanned with the QuantumGX MicroCT imaging system (PerkinElmer, Waltham, MA, USA). The bone mineral density (BMD) and bone volumetric fraction (BV/TV) were further calculated.

### Statistical analysis

Data was presented as mean ± standard deviation (SD) or box-and-whiskers plot, with the ‘box’ depicting the median and the 25th and 75th quartiles and the ‘whisker’ showing the SD. One-way ANOVA was used to evaluate significant differences of results among four groups. Un-paired *t* test was employed to compare the differences between two groups. *P* < 0.05 was considered statistically significant.

## Results

### Phenotype identification of BMSCs

**Fig. 1:**
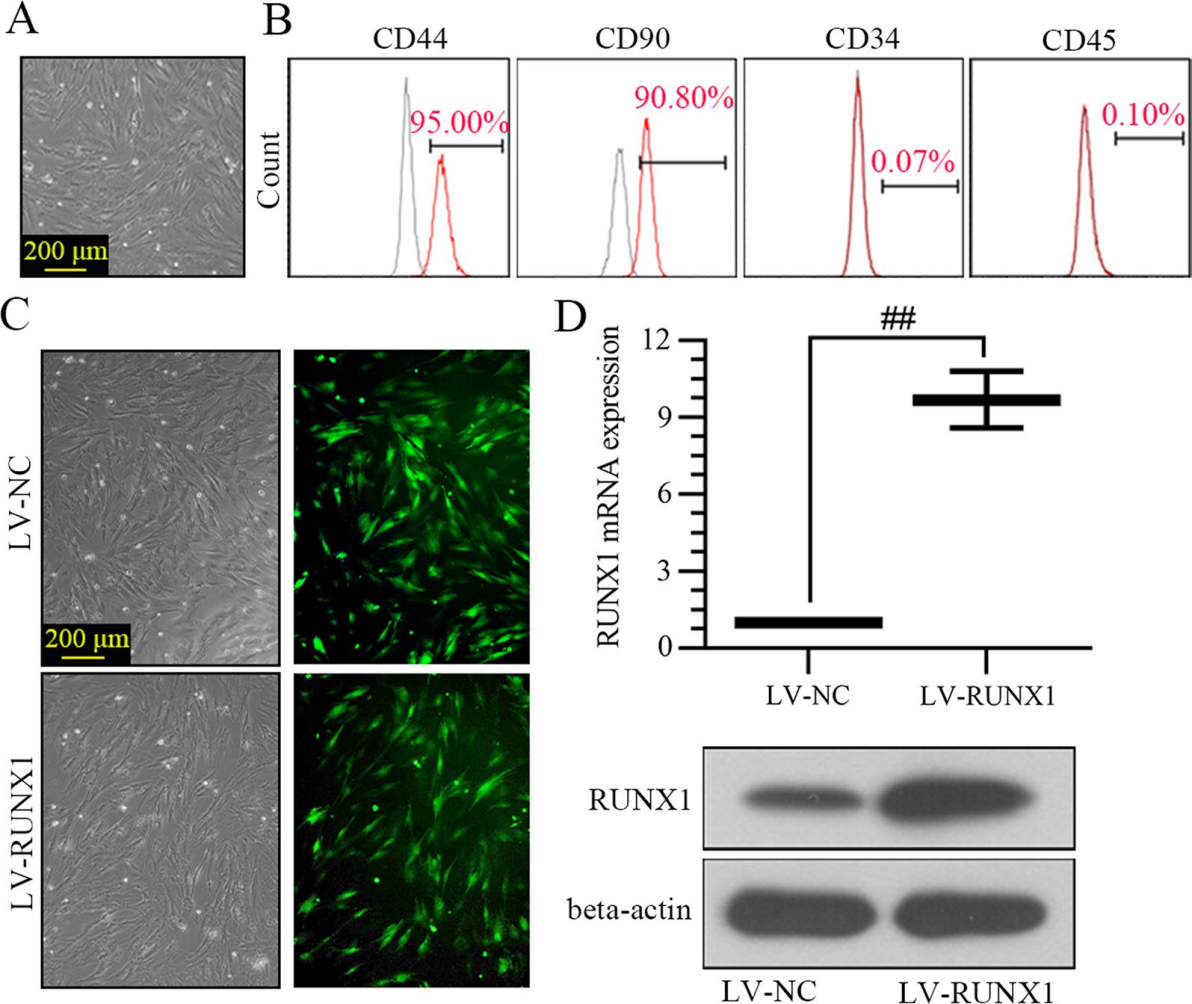
Phenotype identification of BMSCs. **A** Primary BMSCs exhibited a long spindle shape (100× magnification). **B** BMSCs surface antigen identification by flow cytometry. **C** BMSCs infected with LV-NC or LV-RUNX1 by fluorescence microscopy (100× magnification). **D** The mRNA and protein expression of RUNX1 in BMSCs. ^##^*p* < 0.01

As shown in Fig. 1A, cell morphology of BMSCs was diverse, which was testified as sharp boundary and excellent refraction. Expression of cell surface antigens including CD90, CD44, CD34, and CD45 was measured by flow cytometry. Results exhibited positive-antigen CD44 and CD90, as well as negative-antigen CD34 and CD45, suggesting the high purity of the isolated BMSCs (Fig. 1B). Next, BMSCs infected with RUNX1 lentiviral were analyzed for EGFP expression by fluorescence microscopy. High levels of EGFP were detected 72 h post-infection, indicating the high infection efficiency (Fig. 1C). The mRNA and protein expression of RUNX1 was increased in BMSCs infected with LV-RUNX1 compared to LV-NC (Fig. 1D).

### RUNX1-upregulated BMSCs elevates biomechanical strength after ACL reconstruction

**Fig. 2:**
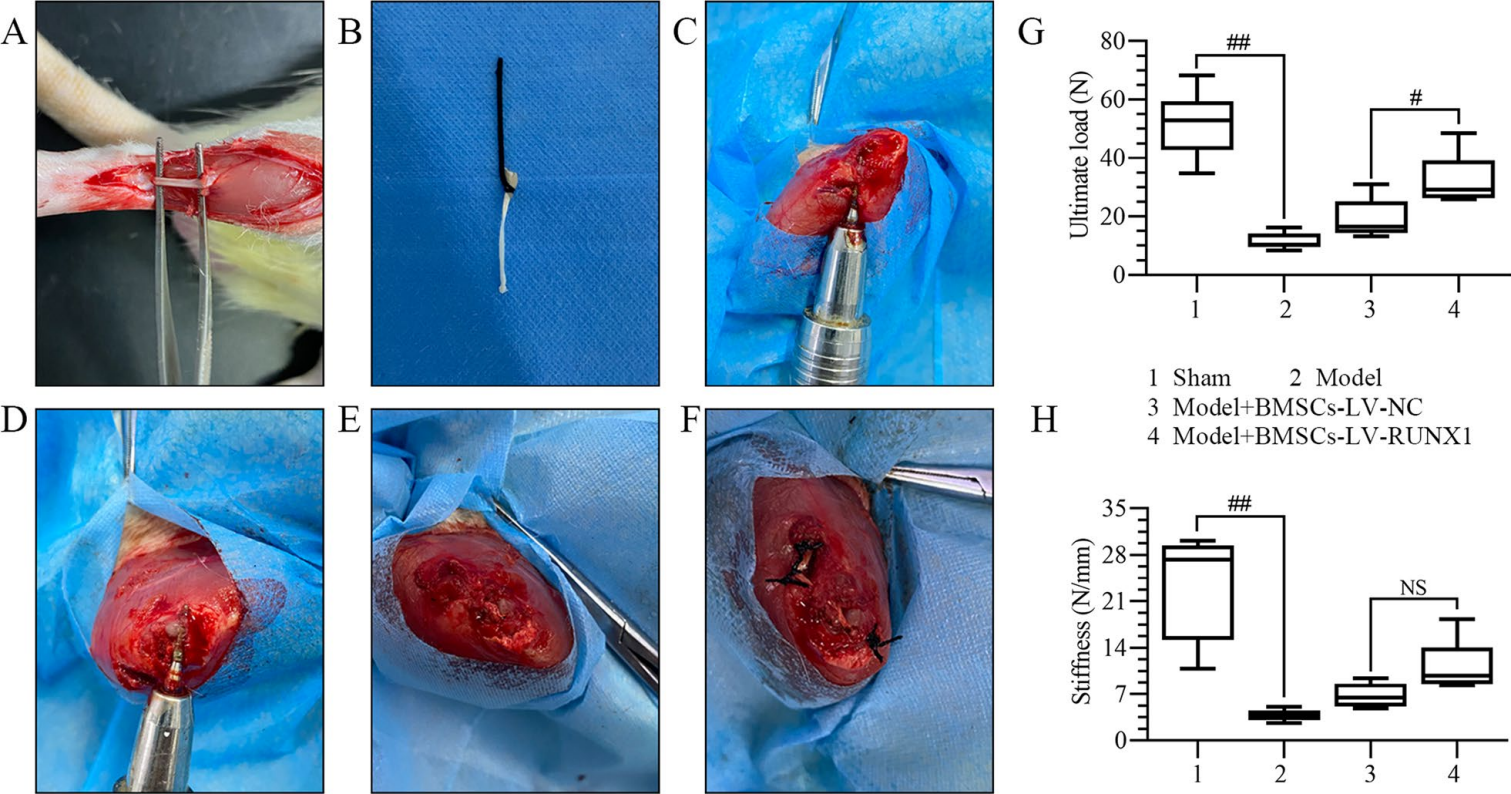
RUNX1-upregulated BMSCs elevates biomechanical strength after ACL reconstruction. **A**–**F** Procedures for applying the BMSCs infected with LV-NC or LV-RUNX1 sheet to the bone tunnel during ACL reconstruction. **A**, **B** Harvest the native tendon as the tendon graft. **C**, **D** Create the femoral and tibial bone tunnels. **E** Insert the tendon graft into both bone tunnels. **F** Fix the tendon graft with suture tied over the neighboring periosteum. **G** Ultimate load of the tendon graft. **H** Stiffness of the tendon graft. ^#^*p* < 0.05, ^##^*p* < 0.01; *NS* not significant

To investigate the effects of RUNX1 on biomechanical strength of tendon–bone junction, a rat model of ACL reconstruction was established. The surgical procedures are shown in Fig. 2A–F and described in detail in the Methods. The biomechanical properties were further explored. As revealed in Fig. 2G, RUNX1-upregulated BMSCs reversed the decrease of ultimate load of the tendon graft complex after ACL reconstruction. The similar trend was observed on stiffness of the tendon graft (Fig. 2H). These results indicated that RUNX1-upregulated BMSCs elevated biomechanical strength after ACL reconstruction.

### RUNX1-upregulated BMSCs accelerates the healing of tendon–bone interface after ACL reconstruction

**Fig. 3:**
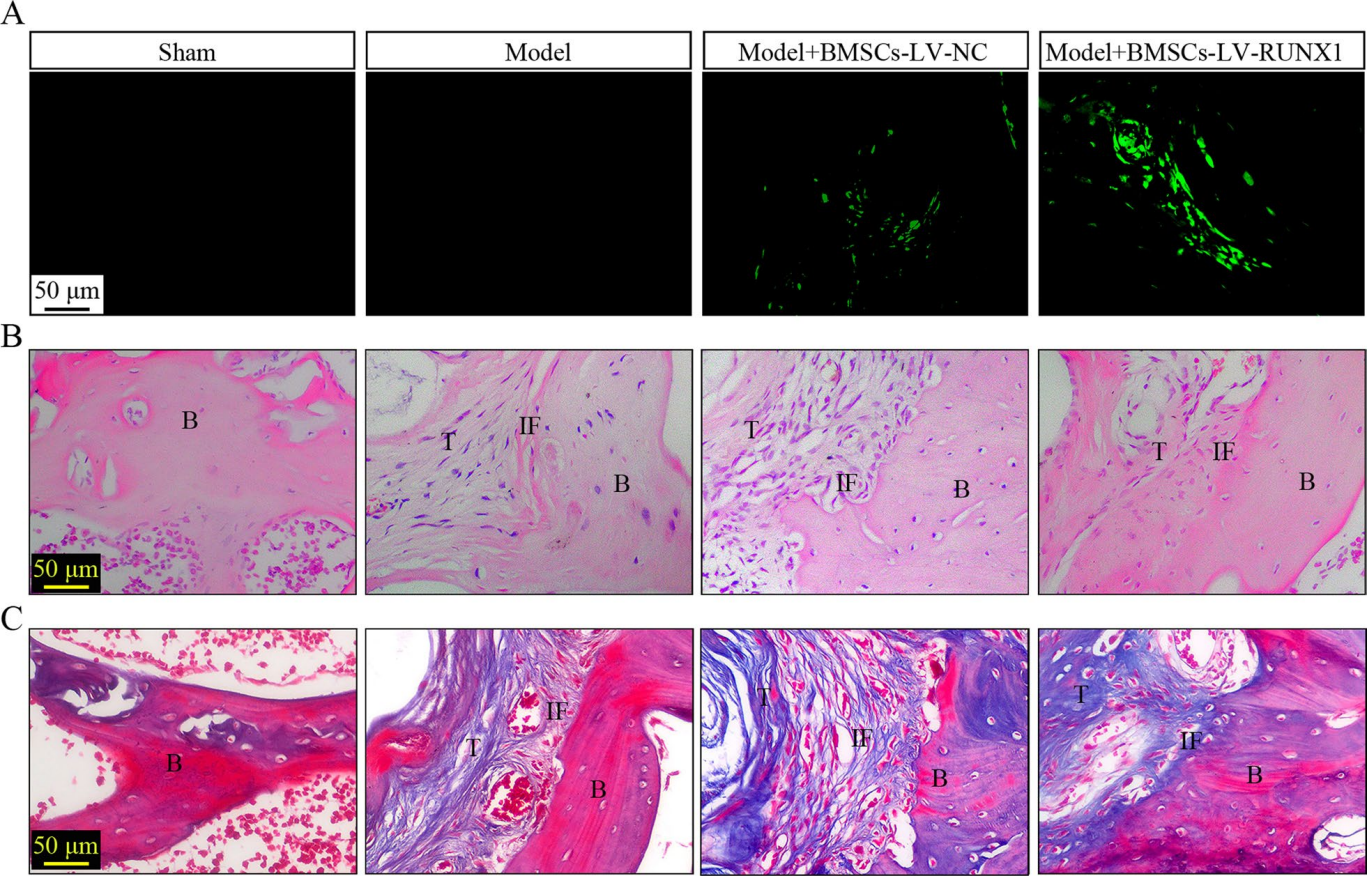
RUNX1-upregulated BMSCs accelerates the healing of tendon–bone interface after ACL reconstruction. **A** Fluorescence imaging of tendon–bone interface (400× magnification). **B** H&E staining of the interface (400× magnification). **C** Masson’s trichrome staining of the interface (400× magnification). *B* bone, *IF* interface, *T* tendon

We further explored the function of RUNX1 on the tendon–bone healing after ACL reconstruction. As shown in Fig. 3A, the number of BMSCs with EGFP expression in tendon–bone interface after ACL reconstruction in LV-RUNX1 group was more than that of LV-NC group. Next, the histological changes were examined by H&E and Masson’s staining. The results demonstrated that upregulation of RUNX1 was linked with better recovery around the bone tunnel, a tighter tendon–bone interface, and more collagen fibers compared to the group of BMSCs infected with LV-NC (Fig. 3B, C). The results indicated the excellent integration and remodeling between the graft and host bone in the RUNX1-upregulated BMSCs group.

### RUNX1-upregulated BMSCs promotes osteogenesis after ACL reconstruction

**Fig. 4:**
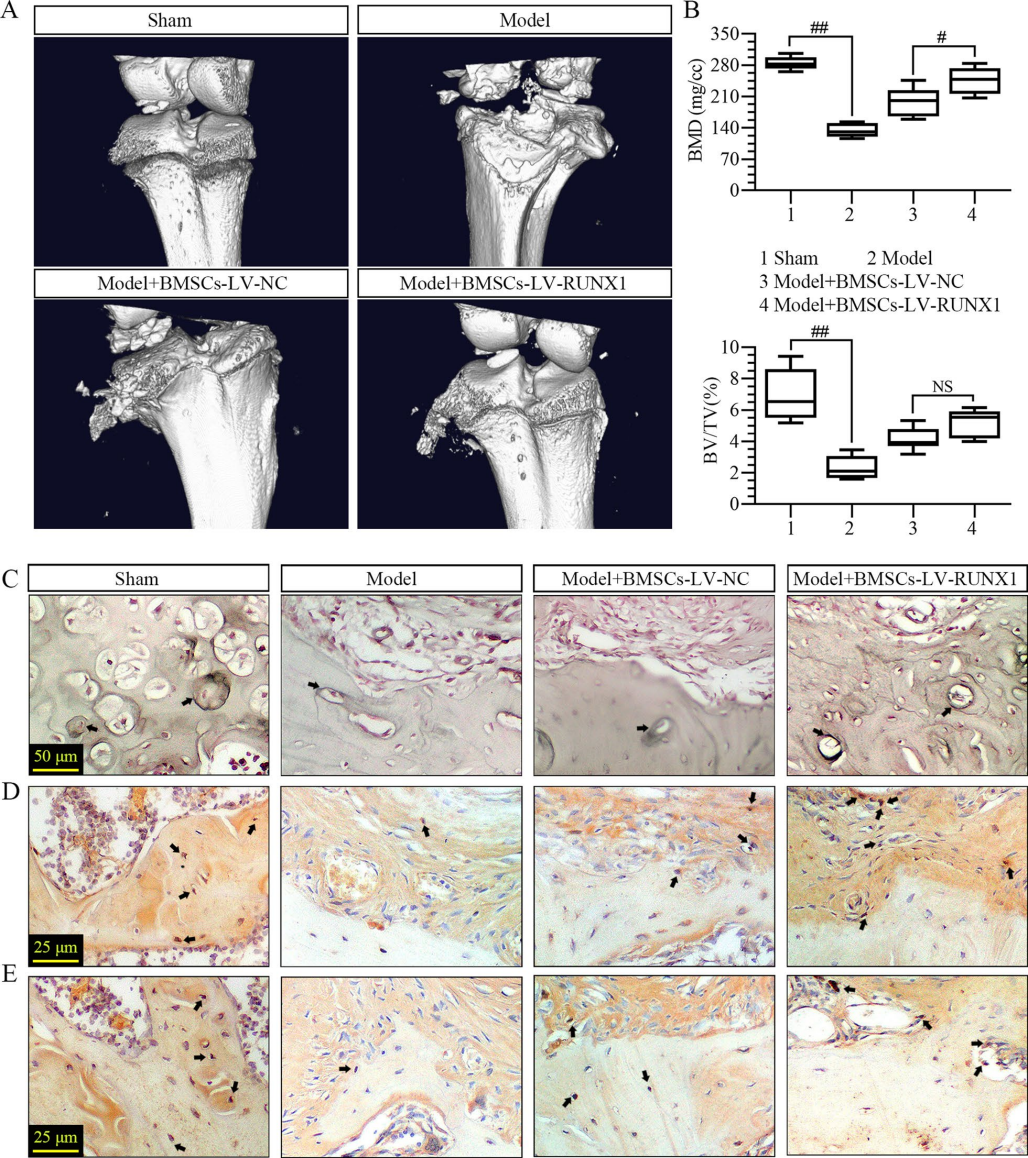
RUNX1-upregulated BMSCs promotes osteogenesis after ACL reconstruction. **A** Micro-CT images of the knee joints. **B** BMD and BV/TV. **C** ALP-stained cells in tendon to bone interface (400× magnification). Black arrows indicated the ALP-positive cells. **D**, **E** The expression of OCN and OPN was assessed by immunohistochemistry (800× magnification). Black arrows indicated the OCN and OPN-positive cells in tendon to bone interface. ^#^*p* < 0.05, ^##^*p* < 0.01; *NS* not significant

We subsequently investigate the effects of RUNX1 on bone formation of tendon–bone interface after ACL reconstruction. Firstly, representative micro-CT 3D images of knee joint of rat were revealed in Fig. 4A. Compared to the specimens in sham group, the surface of the specimens after ACL reconstruction exhibited severe loss and erosion of the cartilage and bone in the tibial area (Fig. 4A). RUNX1 upregulation played a better role in mitigating the damages of the cartilage and bone structure than that of BMSCs infected with LV-NC (Fig. 4A). In addition, quantification of micro-CT images provided evidences that more bone was formed in the RUNX1 upregulation group than in the model group, as evidenced by the increase of the reduced BMD (Fig. 4B). The similar trend was observed in the changes of BV/TV (Fig. 4B). Moreover, we found that the decreased numbers of cells stained with ALP in tendon–bone interface after ACL reconstruction was elevated by the BMSCs with high expression of RUNX1 (Fig. 4C). Then, RUNX1 upregulation rescued the reduced number of OCN and OPN-positive cells in the tendon–bone interface after ACL reconstruction at the presence of BMSCs (Fig. 4D, E). These results indicated that RUNX1-upregulated BMSCs promoted the bone formation after ACL reconstruction.

## Discussion

To our knowledge, this study is the first demonstration of the therapeutic potential of gene therapy using lentivirus-mediated BMSCs expressing RUNX1 for tendon–bone healing after ACL reconstruction. The healing effects were due to the promotion of bone formation in the interface and stronger ultimate load as well as stiffness. These results indicated the protective role of the RUNX1 in the improvement of the regeneration of the tendon–bone interface.

RUNX1, together with RUNX2, and RUNX3 constitute the Runt-related transcription factor family [23]. The three members share highly homology sequence Runt and extensively expressed in various tissues [23]. They act as different parts in the development process. RUNX2 involves in bone formation and is linked with cleidocranial dysplasia [24, 25]. RUNX3 cooperates with RUNX2 to regulate chondrocyte growth and hypertrophy [26, 27]. RUNX1 has been reported to be expressed in different stages of both osteoblast and chondrocyte differentiation [6]. RUNX1 is required for chondrocyte development. Knockdown of RUNX1 causes the delayed endochondral ossification [17], which is vital during lone bone elongation (including tibia and femur) and the healing of bone fractures [28]. Moreover, it has been demonstrated that RUNX1 facilitates the capacity of osteogenesis in BMSCs by repressing adipogenesis through Wnt/β-catenin signaling pathway [18]. Osteoblasts are originated from local mesenchymal stem cells and mediate the process of bone formation. Tendon–bone healing and osteogenesis are essential factors in ACL reconstruction. Previous studies employing BMSCs to enhance the healing of the tendon-to-bone interface revealed that transplantation of BMSCs could promote direct tendon–bone healing to some extent, whereas new osteogenesis is insufficient at the tendon–bone interface [29]. Combined with the potential of RUNX1 on inducing the differentiation from BMSCs to osteoblast, we speculated it may play a role in the recovery after ACL reconstrcution. Using BMSCs as a carrier, the modification of TGF-β1 could facilitate the bone formation and tendon-healing after ACL reconstruction [6]. Herein, the similar results were obtained. Based on the histological observation in the current study, upregulation of RUNX1 was linked with better recovery around the bone tunnel, a tighter tendon–bone interface, and more collagen fibers compared to the group of BMSCs infected with LV-NC.

In addition, the healing of the tendon–bone interface after ACL reconstruction is associated with the changes of mechanical strength. Lim et al. have reported that the maximum loads and stiffness in mesenchymal stem cells-enhanced graft are markedly higher than that of control reconstructions, and this study also demonstrated that mesenchymal stem cells could affect the biomechanical properties of the tendon [30]. Largely consistent with the study, we found that the decreased failure load of graft complex after ACL reconstruction was reversed by RUNX1-upregulated BMSCs compared to BMSCs infected with empty vector. The similar trend was observed in the rigidity. Furthermore, these mechanical properties could be altered by the changes of BMD [31]. A previous study has manifested that BMD acts as an essential part in the strength of tendon graft fixation during ACL reconstruction [32]. Low BMD contributes to fracture [33], suggesting the relation between the elevated BMD and the improvement of the degree of ossification. Yerges et al. have confirmed that single nucleotide polymorphisms in the RUNX1 gene locus are strongly associated with trabecular vertebral BMD in men ages 65 years or older [34]. In this study, quantification of micro-CT images provided evidences that larger bone density was formed in the group of BMSCs with RUNX1 upregulation than in the group of BMSCs infected with LV-NC, as evidenced by the increase of the reduced BMD. The similar trend was observed in the changes of BV/TV.

Moreover, we found that RUNX1 upregulation increased the numbers of ALP, OCN, and OPN-positive cells in the tendon–bone interface after ACL reconstruction. OCN and OPN are the markers of osteogenic differentiation of BMSCs, and ALP is an essential marker of matrix mineralization and osteogenesis [35, 36]. Thus, the results indicated that RUNX1 upregulation promoted the osteogenesis and matrix mineralization of the interface. It has been reported that the mineral and collagen compositions and the structural features of bone matrix determine the biomechanical strength of bone [37]. Together with the results of more collagen fibers in the interface, we preferred that the RUNX1 upregulation-induced matrix mineralization and collagen formation of the tendon to bone interface may lead to the elevation of biomechanical strength.

## Conclusion

RUNX1-upregulated BMSCs contributes to tendon–bone healing after ACL reconstruction, as evidenced by the recovery around the bone tunnel and a tighter tendon–bone interface, as well as the improvement of biomechanical strength and the promotion of osteogenesis.

## Data Availability

The data of this work is available on request to the corresponding author.

## Abbreviations

ACL: Anterior cruciate ligament
ALP: Alkaline phosphatase
BMD: Bone mineral density
BV/TV: Bone volumetric fraction
BMSCs: Bone mesenchymal stem cells
H&E: Haematoxylin and eosin
OCN: Osteocalcin
OPN: Osteopontin
PBS: Phosphate buffer saline
qRT-PCR: Quantitative real-time polymerase chain reaction
RUNX1: Runt related transcription factor 1
SD: Standard deviation
